# Isolation and characterization of a novel human intestinal *Enterococcus faecium* FUA027 capable of producing urolithin A from ellagic acid

**DOI:** 10.3389/fnut.2022.1039697

**Published:** 2022-11-09

**Authors:** Xiaomeng Zhang, Yaowei Fang, Guang Yang, Xiaoyue Hou, Yang Hai, Mengjie Xia, Fuxiang He, Yaling Zhao, Shu Liu

**Affiliations:** ^1^Co-Innovation Center of Jiangsu Marine Bio-industry Technology, Jiangsu Ocean University, Lianyungang, China; ^2^Jiangsu Key Laboratory of Marine Bioresources and Environment/Jiangsu Key Laboratory of Marine Biotechnology, Jiangsu Ocean University, Lianyungang, China; ^3^School of Food Science and Engineering, Jiangsu Ocean University, Lianyungang, China; ^4^Key Laboratory of Marine Drugs, School of Medicine and Pharmacy, The Ministry of Education of China, Ocean University of China, Qingdao, China

**Keywords:** ellagic acid, urolithin A, *Enterococcus faecium*, novel probiotic, metabolism

## Abstract

Urolithin A (UA) has received considerable research attention because of its health benefits. However, only a few strains have been reported to produce UA from ellagic acid (EA), and the molecular mechanisms underlying the gut microbiota-mediated transformation of ellagic acid into urolithin A is limited. In the present study, a single strain FUA027 capable of converting ellagic acid into UA *in vitro* was isolated from the fecal samples. The strain was identified as *Enterococcus faecium* through the morphological, physiological, biochemical and genetic tests. UA was produced at the beginning of the stationary phase and its levels peaked at 50 h, with the highest concentration being 10.80 μM. The strain *Enterococcus faecium* FUA027 is the first isolated strain of *Enterococcus sp.* producing urolithin A from ellagic acid, which may be developed as probiotics and used to explore molecular mechanisms underlying the biotransformation of ellagic acid into UA.

## Introduction

A healthy diet not only supports the energy and physiological needs of the body even in the absence of excess food intake but also prevents diseases and positively influences health (1), the role of microbiota in health and diseases is also being highlighted (2). This point is supported by scientific evidence obtained by investigating foods rich in ellagic acid and ellagitannin (3). Ellagic acid is a polyphenol and a natural antioxidant widely found in many vegetables, fruits, nuts, and teas (4, 5). However, their health applications have been limited due to their poor water solubility and low bioavailability (6). Only a small amount of this polyphenol is absorbed by the epithelial cells of the small intestine (7). The first reported urolithins were urolithin A and urolithin B that were found in kidney stones of sheep. Besides, the intestinal flora of human and animals also transforms some of the unabsorbed ellagic acid or ellagitannin into urolithins, mainly including isourolithin, urolithin A, urolithin B, and urolithin C. Urolithins, which are more easily absorbed than their precursors, have been widely investigated and have exhibited various biological activities (4, 8), such as anticancer, antioxidative (9), anti-inflammatory, neuroprotective, antiaging, and anti-obesity activities (10). Significantly, urolithin A can improve mitochondrial function, which is the important characteristic of urolithin A compared with other intestinal metabolites.

Urolithin A does not exist in the natural state, which can be produced by the metabolism of ellagic acid through intestinal microorganisms of human. The bioconversion capacity of ellagitannin/ellagic acid to urolithins varies from person to person (11). People are classified into three urolithin metabotypes on the basis of urolithins metabolized by their intestinal microbiota, namely UM-A, UM-B, and UM-0 (12). UM-A only produces urolithin A; UM-B is characterized by urolithin B and isourolithin A, in addition to a low concentration of urolithin A, whereas UM-0 does not produce these urolithins. Urolithin A was one of the most widely studied urolithins, which reduced triglyceride accumulation in the liver (13). Moreover, it can inhibit inflammatory factors, such as TNF-α, IL-1β, it played an important role in the treatment of various inflammations (13). Urolithin A exhibited stronger anti-inflammatory activity than urolithin B. Urolithin A stimulates mitophagy and improves muscle health in both mice and humans (14, 15), and urolithin A prevents the accumulation of dysfunctional mitochondria to extends the lifespan of the nematode *Caenorhabditis elegans* (16–18). Moreover, urolithin A was shown to be safe by a clinical study, and it improved exercise capacity in mouse models of age-related decline of muscle function. Based on the bioavailability of urolithin A in muscle, the preventive effect of urolithin A treatment on age-related muscle decline in 16-month-old male C57BL/6J mice fed a high-fat diet (HFD) was evaluated by Ryu, D., et al. From the age of 16 months, the diet was supplemented with 50 mg/kg/d urolithin A for 8 months. Urolithin A treatment resulted in a significant increase in muscle function measured at 22 and 24 months of age compared to control HFD-fed mice (18). Consequently, urolithin metabotypes affect the metabolism and bioactivity of dietary polyphenols including ellagic acid and ellagitannins, thereby making them exhibit different effects on people’s health (17).

Although several studies have demonstrated that the gut microbiota composition is a critical factor in the bioconversion of ellagic acid and ellagitannins to urolithins (19), the mechanism underlying this transformation is unclear. This would help us maximize the biological activitiy of urolithins, especially for the metabotypes UM-0 and UM-B (12, 20). In 2020, Amazentis sold high-purity urolithin A as a nutritional product for improving muscular strength and fighting aging. So far, chemical synthesis is the only method for producing urolithin A for industrial purposes. Compared with chemical synthesis, biosynthesis of urolithin A is an economical and environmentally friendly method (21). Therefore, screening strains metabolizing ellagitannin/ellagic acid to urolithin A and exploring their metabolite regulation are necessary (22). However, intestinal bacteria with the ability to transform ellagic acid into urolithin A have rarely been reported. To date, *Gordonibacter urolithinfaciens* DSM 27213T = CCUG 64261T, *Gordonibacter pamelaeae* DSM 19378T = CCUG 55131T (23, 24), *Ellagibacter isourolithinifaciens* DSM 104140T = CCUG 70284T (12, 25), and *Bifidobacterium pseudocatenulatum* INIA P815 have been reported to produce urolithins from ellagic acid (26). Among them, only *B. pseudocatenulatum* INIA P815 showed urolithin A-producing ability (26).

In the present study, a urolithin A-producing strain, designated FUA027, was isolated from human feces. The strain was identified as *Enterococcus faecium* on the basis of the results of the morphological, physiological, biochemical and genetic tests. The time course of the strain-mediated transformation of ellagic acid into urolithin A was also determined through high-performance liquid chromatography (HPLC) and ultra-performance liquid chromatography-tandem mass spectrometry (UPLC-MS) (23, 27).

## Materials and methods

### Chemicals

L-Cysteine hydrochloride, urolithin A, urolithin B, urolithin C, isourolithin A, urolithin M6, and urolithin D were purchased from Dalton Pharma Services (Toronto, Canada). Ellagic acid was purchased from Sigma-Aldrich (St. Louis, MO). MS- or LC-grade solvents, namely methanol, acetonitrile, formic acid, and ethanol were purchased from Merck (Darmstadt, Germany).

### Collection of human fecal samples

All 7 volunteers (ages 22–27) with no history of gastrointestinal diseases signed an experimental informed consent form at the Jiangsu Ocean University (Lianyungang, China). The volunteers had not used antibiotics 3 months before the study. After took 0.7 g food/kg body weight/day for 2 consecutive weeks, feces samples were collected at 6:30 AM on the 15th day before they ate any food. Within 10 min, the samples were used for the fermentation experiments described below (24, 28).

### Screening of human fecal samples converting ellagic acid into urolithin A *in vitro*

In total, 10 g of fecal samples were diluted 1/10 (w/v) with anaerobe basal broth (ABB, Oxoid, Basingstoke, England) and homogenized using a stomacher in filter bags (29, 30). 1 ml of filtered fecal suspensions were inoculated into 100 ml of ABB medium supplemented with 20.00 μM ellagic acid and 0.05% L-cysteine hydrochloride and cultured for 5 days in an anaerobic chamber (Don Whitley Scientific Limited, West Yorkshire, England) with an atmosphere consisting of N_2_/H_2_/CO_2_ (80:10:10) at 37°C. Ellagic acid was first dissolved in polyethylene glycol (PEG) 300. The resulting solution was added to ABB medium 20.00 μM. In addition, media without fecal suspension and those without ellagic acid were used as controls. Samples (5 ml) were collected once per day from each culture, extracted with 5 ml C_2_H_3_N:H_2_O:CH_2_O_2_ (80:19.9:0.1), and further analyzed using High Performance Liquid Chromatography (HPLC), as explained below. If urolithin A was detected in the broth, it was used to screen urolithin A-producing bacteria. ABB medium (g/L): Peptone 16.0, Yeast extract 7.0, NaCl 5.0, Starch 1.0, Glucose 1.0, C_3_H_3_NaO_3_ 1.0, Arginine 1.0, C_4_H_4_Na_2_O_4_ 0.5, L-Cysteine HCl 0.5, NaHCO_3_ 0.4, Fe_4_(O_7_P_2_)_3_ 0.5, C_34_H_32_ClFeN_4_O_4_ 0.005,Vitamin K 0.0005, C_2_H_3_NaO_2_S 0.5, C_4_H_10_O_2_S_2_ 1.0,pH6.8 ± 0.2.

### Screening of urolithin A-producing bacteria from the fermentation broth

The urolithin A-containing fermentation broth was diluted to 10^–3^ to 10^–6^ with ABB media. An aliquot of the diluted suspension (0.1 ml) was spread on the ABB agar plate, and the plate was incubated at 37°C in an anaerobic chamber under the condition of N_2_/H_2_/CO_2_ (80:10:10, V:V:V). Selected single colonies were inoculated into 50 ml of ABB containing 20 μM ellagic acid (31). After anaerobic incubation at 37°C for 4 days, the broths were extracted with 50 ml C_2_H_3_N:H_2_O:CH_2_O_2_ (80:19.9:0.1, V:V:V) and further analyzed through HPLC and liquid chromatography-tandem mass spectrometry (LC-MS/MS) analyses, explained below. The urolithin A-producing strains were stored in ABB media with 20% (v/v) glycerol at –80°C for further experiments.

### Identification and taxonomical studies of strain FUA027

The morphological, physiological, and biochemical properties of strain FUA027 were investigated using previously described methods (32). Genomic DNA was extracted using a DNA extraction kit (TIANamp Bacteria DNA Kit, Beijing Tiangen Biotechnology Co., Ltd.). The 16S rDNA sequence was amplified through a PCR procedure by using the universal primers (27F 5’-AGAGTTTGATCCTGGCTCAG-3’, 1492R 5’-TACGACTTAACCCCAATCGC-3’) (33). The PCR product was purified and sequenced. A similarity search in the EMBL database was performed using the BLAST program (National Center for Biotechnology Information). Sequence alignment was executed using the Clustal W algorithm, and a neighbor-joining phylogenetic tree was constructed using MEGA 11 software.

### Growth kinetics and time-course transformation of ellagic acid into urolithin A by strain *Enterococcus faecium* FUA027

*Enterococcus faecium* FUA027was streaked to single colonies on an ABB agar plate at 37°C for 6 days. A liquid culture for the inoculum was prepared by transferring a single colony to a tube containing 5 ml ABB by using a sterile inoculation loop. Two milliliters of the inoculum were transferred to 100 ml ABB media containing 20 μM ellagic acid and incubated under anaerobic conditions consisting of N_2_/H_2_/CO_2_ (80:10:10, V:V:V) at 37°C for 7 days. During incubation, 5-ml aliquots were taken and extracted with 5 ml of C_2_H_3_N:H_2_O:CH_2_O_2_ (V:V:V, 80:19.9:0.1) for HPLC and UPLC-MS analyses as described below. Similarly, culture samples were collected every 2 h on all 7 days of incubation (34), and the cell density was measured at 600 nm using a microplate reader (SpectraMax 190, Molecular Devices, Sunnyvale, CA, USA), the experiment was repeated three times. Data were subjected to statistical analysis of standard deviation. The quantitative estimates represent mean values ± standard error.

### High-performance liquid chromatography analyses

The extracted samples were evaporated to dryness under a N_2_ stream at room temperature. The resultant extract was dissolved in 500 μl methanol/water (50:50, v/v). A 0.22-μm cellulose acetate filter (Millipore, Madrid, Spain) was used to filter the extract. The HPLC analysis was performed on a ZORBAX SB-C18 column (250 mm × 4.6 mm, 5 μm particle size, Agilent Technologies) at 25°C. The gradient started at 20% A and 80% B, followed by 70% A and 30% B at 15 min, 95% A and 5% B at 20 min, 100% A and 0% B at 21 min, 100% A and 0% B at 23 min, 20% A and 80% B at 24 min, and 20%A and 80% B at 25 min. The flow rate was 1 ml/min, and the column temperature was 25°C. Injection volume was 5 μl for each sample. UV chromatograms were recorded at 305 nm.

### Liquid chromatography–mass spectrometry/mass spectrometry analyses

The Q-Exactive LC-MS/MS (Thermo Fisher) was used to analyze urolithin A using a C18 column [(Waters Ltd., MA, USA) ACQUITY UPLC^®^ BEH C18, 2.1 × 50 mm, 1.7 μm; 0.5 ml/min] and HR-ESI-MS scan range from 150.0 to 2,000.0 Da. Sheath gas flow rate, 45 arb; aux gas flow rate; 15 arb; Capillary temp:320°C; S-Lens RF Level 50 T. chromatographed using LC-MS/MS at 30°C. The mobile phase consisted of solvent A (water, 0.1% formic acid) and solvent B (acetonitrile). Separation was performed at a flow rate of 0.5 ml/min with the following gradient elution: 0–10.5 min, a linear gradient runs from 10 to 100% solvent B; 10.5–12.5 min, 100% solvent B; 12.5–12.6 min, a linear gradient runs from 100 to 10% solvent B; 12.6–16.5 min 10% solvent B for re-equilibration. The limits of detection (LODs) were determined based on a signal-to-noise ratio (S/N) of 3 and of 10 for the limit of quantification (LOQ). The LODs: 0.5 μM (EA and urolithin A), 0.2 μM (urolithin C), the dynamic range: 0.5–20 μM. Urolithin A and urolithin C in the fermentation broth were first identified through HPLC and further identified by comparing the molecular mass of the compound obtained with that of a pure standard by using LC-MS/MS (35, 36).

## Results and discussion

### Screening of human fecal samples converting ellagic acid into urolithin A *in vitro*

**FIGURE 1:**
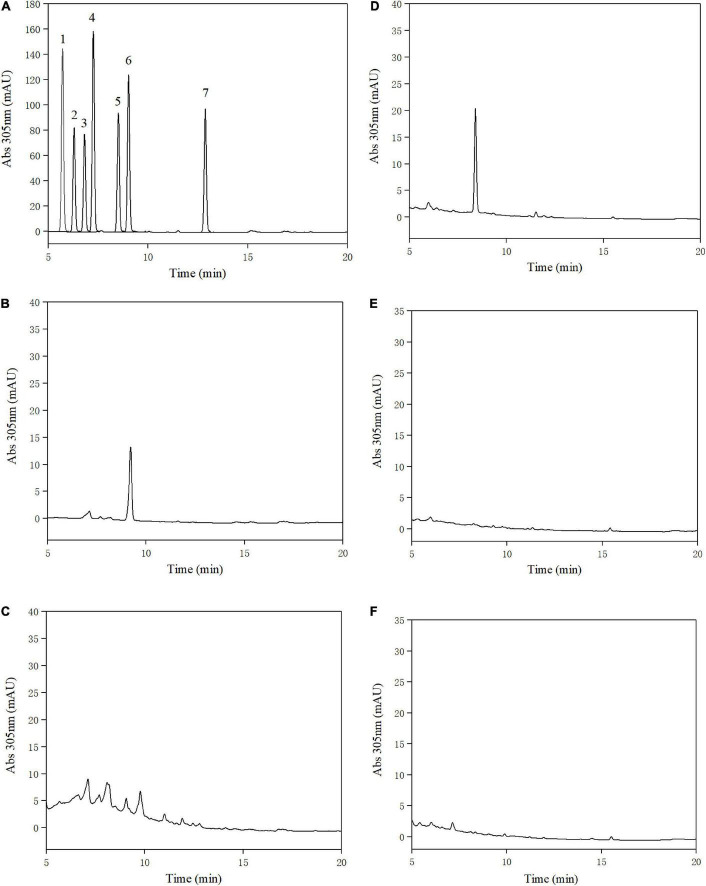
High-performance liquid chromatography (HPLC) analysis of urolithins obtained and standard samples at 305 nm. Standard samples of urolithins: 1. urolithin D, 2. ellagic acid, 3. urolithin M6, 4. urolithin C, 5. isourolithin A, 6. Urolithin A, 7. urolithin B **(A)**, fecal samples of volunteer L **(B)**, volunteer X **(C)**, volunteer H **(D)**, medium without fecal suspension **(E)**, medium without ellagic acid **(F)**.

Urolithins were identified through a direct comparison of the retention times with those of pure standards (10). The retention times of standards were analyzed by the HPLC at absorbance of 305 nm. The results revealed that urolithin A was produced by three human fecal samples, which indicates that three volunteers were metabotype A (Figure 1) (the concentration of urolithin A: volunteer L, 2.15 μM; volunteer X, 0.56 μM; volunteer H, 3.32 μM). A similar result was reported by Cortés-Martín et al., indicating that aging is a key factor that influences the distribution of urolithin metabotypes (11).

### Screening of urolithin A-producing bacteria

**FIGURE 2:**
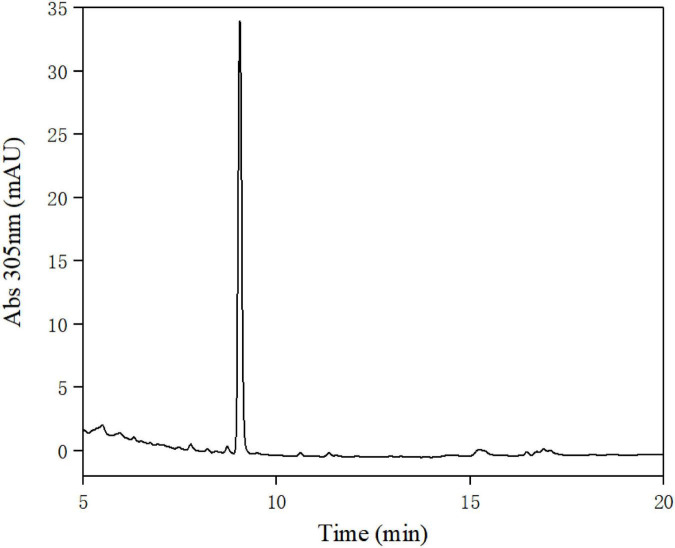
High-performance liquid chromatography (HPLC) analysis of the extract of the fermentation broth of strain FUA027.

**FIGURE 3:**
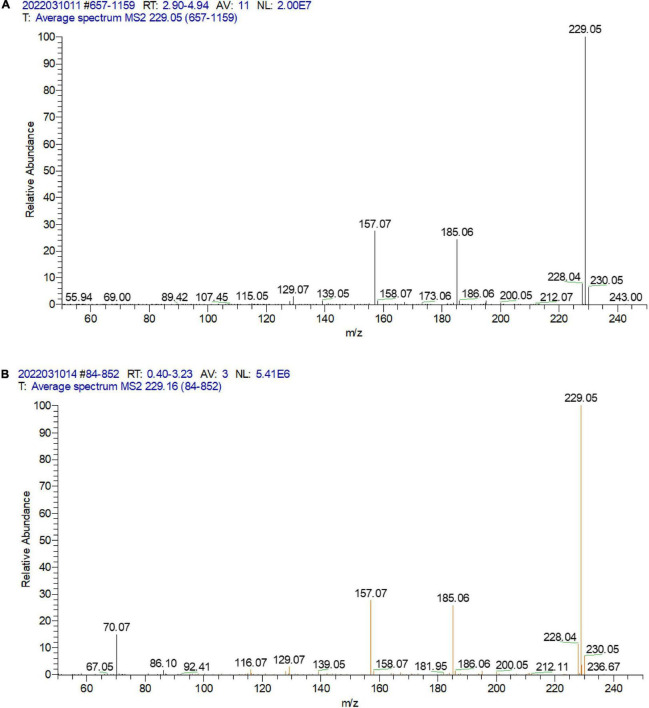
LC-MS/MS analysis of the standard urolithin A **(A)** and the urolithin A of the extract of the fermentation broth of strain FUA027 **(B)**.

A total of 395 strains were screened for urolithin A production. According to the results of HPLC analysis (Figure 2) and LC-MS/MS (Figure 3), only FUA027 strain can convert ellagic acid into urolithin A under anaerobic conditions. Several reports have shown that the specific intestinal microflora of some people can transform ellagic acid/ellagitannins consumed into urolithin A (37). The species or composition of gut flora that produce urolithin A has not, however, been investigated. Although urolithin A was found in humans nearly 40 years ago, the pathway for its transformation remains unknown (23). Therefore, to completely comprehend the molecular mechanism of strains or flora underlying the transformation of ellagic acid into urolithins, strains capable of producing urolithin A should be screened and investigated (38). Moreover, probiotics must be developed to increase the urolithin A production efficiency of low-yield individuals (37, 39).

### Identification of the urolithin A-producing strain FUA027

**FIGURE 4:**
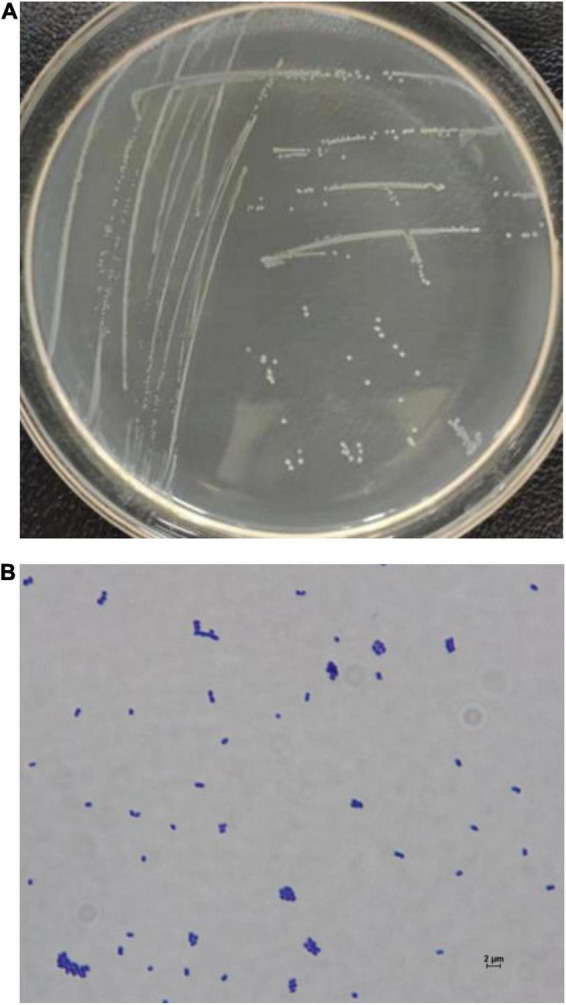
The colony morphology of the strain FUA027 on the ABB plate **(A)**, light microscopic picture (40 × 100 magnification) **(B)**.

**FIGURE 5:**
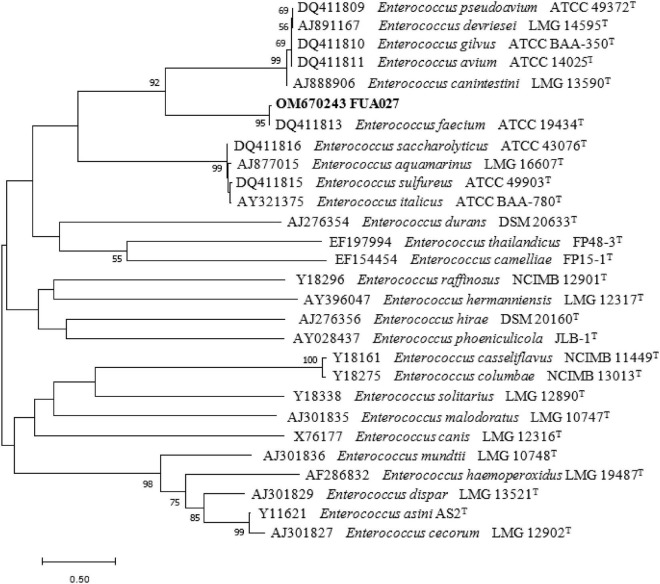
Phylogenetic tree of strain FUA027 within the genus *Enterococcus* based on 16S rDNA sequences. The branching pattern was generated by using the maximum-likelihood method. Based on 100 replications, bootstrap percentages >50% are shown. The bar below the tree represents 1 nucleotide changes per 1,000 nucleotides.

The cells of FUA027 strain were gram-positive and rod-shaped (Figure 4). Simultaneously, based on the comparison of the results of physiological and biochemical measurements with the identification standards (32, 40, 41), strain FUA027 was preliminarily identified as *E. faecium* (Table 1). The 16S rDNA sequence was submitted to GenBank (Accession No.: OM670243). The phylogenetic tree, which was constructed for comparing the 16S rDNA sequences, suggested that strain FUA027 belongs to the genus *Enterococcus* (Figure 5). The closest relative of this strain was strain ATCC 19434^T^ with a valid published name of *E. faecium* (99.36% similarity). Therefore, strain FUA027 was identified as *E. faecium*.

**TABLE 1:** Morphological, physiological, and biochemical characteristics of strain FUA027.

Properties	Result
Shape	Coccus
Gram stain	+
Motility	–
45°C	+
3% NaCl	+
pH 9.0	+
Catalase	–
H_2_S production	–
Nitrate reduction +	–
Methyl red test	–
Vopes-prokauer test	+
Esculin	+
Salicin	+
Cellobiose	+
Galactose	+
Lactose	+
Sucrose	+
Fructose	+
Maltose	+
Mannitol	+
Raffinose	–
Rhamnose	–
Sorbitol	–
Xylose	–
Synanthrin	+

A balance of intestinal flora is beneficial for the health of organisms, conducive to biological activity (7). *E. faecium* is a part of the normal flora of human and animal intestines. When *E. faecium*, harmful intestinal bacteria in humans (*Escherichia coli*), and human intestinal probiotics (*Bifidobacterium*) were cultured, *E. faecium* was found to inhibit these harmful bacteria along with *Bifidobacterium*. *E. faecium* rapidly adheres to the intestinal mucosa after entering the intestine, forming an intestinal barrier (42, 43) and inducing the production of cytokines, interferons, and interleukins to enhance the body’s nonspecific immunity and improve disease resistance. Thus, *E. faecium* can be developed into probiotics (40, 44). The strain FUA027 was deposited in China General Microbiological Culture Collection Center (CGMCC) with accession number 24964.

### Growth kinetics and time-course transformation of ellagic acid into urolithin A by *Enterococcus faecium* FUA027

**FIGURE 6:**
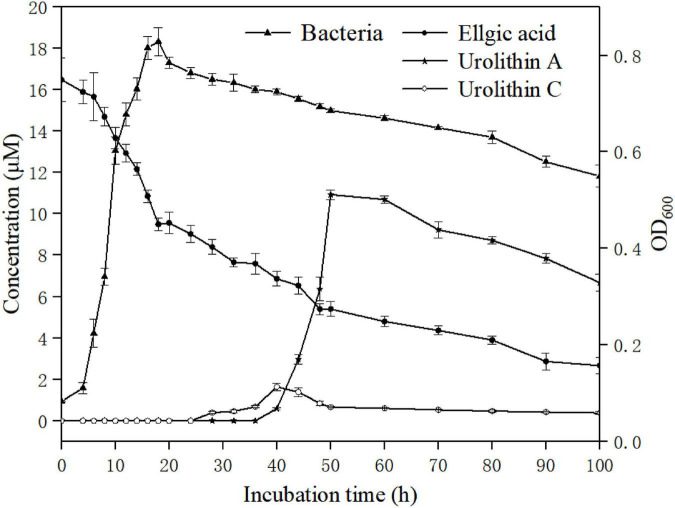
Bacterial growth and time course of urolithin production by the strain FUA027. Ellgic acid (solid circles); Urolithin A (solid stars); Urolithin C (open circles); Bacterial growth curve (solid triangles).

The growth curves of strain FUA027 and its associated transformation of ellagic acid into urolithin A are presented in Figure 6. The lag phase was 5.67 ± 0.78 h, and the growth rate was 0.26 ± 0.04/h in the presence of ellagic acid. Ellagic acid catabolism and urolithin formation occur during the early stage of the mid-logarithmic growth phase of *E. faecium* FUA027. The first metabolite produced after incubating ellagic acid with *E. faecium* FUA027 was urolithin C (thihydroxy-urolithin), which was observed at 28 h and whose levels peaked at 40 h, with the maximum concentration being 1.54 μM. Then, the urolithin C levels declined progressively as urolithin A was formed (Figure 6). Urolithin A was detected after 40 h of incubation, and a maximum urolithin A concentration of 10.80 μM was attained at 50 h. Then, the urolithin A levels decreased gradually (Figure 6). Ellagic acid (20.00 μM) was finally converted to urolithin A (6.44 μM). The urolithin A levels decreased gradually is a phenomenon that can’t be fully explained. Further research is necessary to better understand the reasons of phenomenon.

To date, four urolithin-producing strains have been reported; however, they differ from one another in ellagic acid biotransformation processes. *Gordonibacter* was the first reported genus to transform ellagic acid into urolithins, producing urolithin M5, urolithin M6, and urolithin C. Ellagic acid catabolism and urolithin formation occurred during the stationary growth phase of *G. urolithinfaciens* and *G. pamelaeae*. Urolithin C was the end product of ellagic acid transformation by the two strains (23, 24). Ellagic acid and urolithin C catabolism also occurred during the stationary growth phase in *E. isourolithinifaciens*, but isourolithin A was the end product of ellagic acid catabolism (25). The end products of ellagic acid catabolism by strain *B. pseudocatenulatum* INIA P815 were urolithins A and B. The transition from ellagic acid to the intermediary urolithins was rapid; however, only urolithins A and B, but not the intermediary urolithins, were detected (26). Similar results have been reported by García-Villalba et al. (34). The conversion rate of urolithin A in a single bacterium (*Bifidobacterium pseudocatenulatum* INIA P815) reported in the literature is less than 2% (26). Compared with the reported strain, the conversion rate of urolithin A in a single bacterium (*E. faecium* FUA027) is 32.2%, the conversion rate was higher than the reported strain significantly. FUA027 is the great candidate strain to study the mechanism underlying the biotransformation of ellagic acid into urolithin A.

## Conclusion

The transformation of ellagic acid into urolithin A by a single bacterial strain is described herein. This is the first description of an *E. faecium* strain capable of converting ellagic acid into urolithin A *in vitro*. The strain *E. faecium* FUA027 could potentially be useful for the development of next-generation probiotics, functional foods, and food complements and be used to explore the regulation of their transcription.

## Data availability statement

The original contributions presented in this study are included in the article/supplementary material, further inquiries can be directed to the corresponding author.

## Author contributions

XZ: conceptualization, methodology, software, and writing – original draft. YF: resources and supervision. GY: conceptualization and resources. XH: data curation and supervision. YH: software and validation. MX and YZ: investigation. FH: formal analysis. SL: methodology, supervision, and project administration. All authors contributed to the article and approved the submitted version.
